# 2,2′-Dimethyl-4,4′-bipyridine

**DOI:** 10.1107/S1600536808017807

**Published:** 2008-06-19

**Authors:** Bahtier Ibragimov, Edwin Weber, Max Peukert, Conrad Fischer, Wilhelm Seichter

**Affiliations:** a Institute of Bioorganic Chemistry, Academy of Sciences of Uzbekistan, H. Abdullaev 83, Tashkent 100125, Uzbekistan; b Institut für Organische Chemie, TU Bergakademie Freiberg, Leipziger Strasse 29, D-09596 Freiberg/Sachsen, Germany

## Abstract

In the crystal structure of the title compound, C_12_H_12_N_2_, the mol­ecule is twisted around the central C—C bond, with a dihedral angle of 8.32 (5)° between the mean planes of the pyridyl rings. The crystal structure is stabilized by arene stacking inter­actions, with a distance of 3.81 (1) Å between the ring centroids.

## Related literature

For related literature, see: Boag *et al.* (1999 ▶); Kraft *et al.* (2005 ▶); Leighton & Sanders (1987 ▶); Alcade *et al.* (2007 ▶); Boghala *et al.* (2005 ▶); Braunschweig *et al.* (2006 ▶); Diskin-Posner *et al.* (2005 ▶); Kryschenko *et al.* (2003 ▶); Lynch *et al.* (1999 ▶); Yaghi *et al.* (1995 ▶).
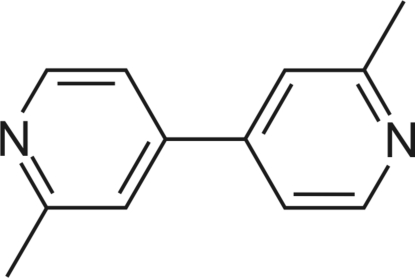


## Experimental

### 

#### Crystal data

C_12_H_12_N_2_
*M* *_r_* = 184.24
Orthorhombic, 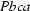

*a* = 11.7961 (3) Å
*b* = 7.6130 (2) Å
*c* = 21.2977 (5) Å
*V* = 1912.61 (8) Å^3^
*Z* = 8
Mo *K*α radiation
μ = 0.08 mm^−1^
*T* = 153 (2) K
0.54 × 0.24 × 0.14 mm

#### Data collection

Bruker X8 APEXII CCD area-detector diffractometer
Absorption correction: multi-scan (*SADABS*; Sheldrick, 2003 ▶) *T*_min_ = 0.923, *T*_max_ = 0.989
26242 measured reflections
3233 independent reflections
2262 reflections with *I* > 2σ(*I*)
*R*_int_ = 0.066

#### Refinement

*R*[*F* ^2^ > 2σ(*F* ^2^)] = 0.045
*wR*(*F* ^2^) = 0.139
*S* = 0.91
3233 reflections
129 parameters
H-atom parameters constrained
Δρ_max_ = 0.37 e Å^−3^
Δρ_min_ = −0.26 e Å^−3^

### 

Data collection: *APEX2* (Bruker, 2004 ▶); cell refinement: *SAINT-NT* (Bruker, 2004 ▶); data reduction: *SAINT-NT*; program(s) used to solve structure: *SHELXS97* (Sheldrick, 2008 ▶); program(s) used to refine structure: *SHELXL97* (Sheldrick, 2008 ▶); molecular graphics: *ORTEP-3* (Farrugia, 1997 ▶); software used to prepare material for publication: *SHELXTL* (Sheldrick, 2008 ▶).

## Supplementary Material

Crystal structure: contains datablocks global, I. DOI: 10.1107/S1600536808017807/cs2078sup1.cif

Structure factors: contains datablocks I. DOI: 10.1107/S1600536808017807/cs2078Isup2.hkl

Additional supplementary materials:  crystallographic information; 3D view; checkCIF report

## Figures and Tables

**Table 1:** Hydrogen-bond geometry (Å, °)

*D*—H⋯*A*	*D*—H	H⋯*A*	*D*⋯*A*	*D*—H⋯*A*
C6—H6*C*⋯N1^i^	0.98	2.73	3.6728 (16)	161

Symmetry code: (i) 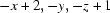.

**Fig. 1.**
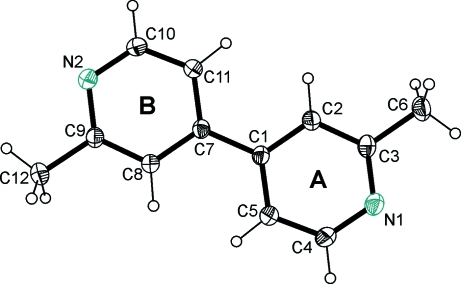
Molecular presentation of the title compound with atomic labels and 50% probability displacement ellipsoids for non H-atoms.

**Fig. 2.**
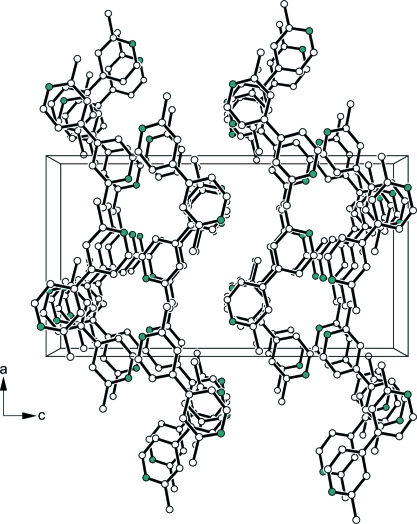
Packing diagram as viewed down the crystallographic b axis.

## supplementary crystallographic information

### Comment

The preparation of I was carried out according to the literature procedure (Leighton & Sanders, 1987). The title compound represents a derivative of 4,4'-bipyridine, which is widely used as a bifunctional bridging element for the synthesis of supramolecular assemblies which may be based on hydrogen bond interactions (Lynch *et al.*, 1999, Boghala *et al.*, 2005) or metal coordination complexes (Diskin-Posner *et al.*, 2005, Kryschenko *et al.*, 2003), involving catenanes (Alcade *et al.*, 2007), rotaxanes (Braunschweig *et al.*, 2006) and metal-organic frameworks (Yaghi *et al.*, 1995). In the crystal the title molecule adopts a slightly twisted conformation (see Figure 1), the mean planes of the hetero - aromatic rings make 8.32 (5)° dihedral angle. As there are no good hydrogen bond donors, the N1 nitrogen atom is only involved in the formation of a weak C-H···N hydrogen bond [C6-H6C···N1 distance ca. 2.73 Å]. The packing (Figure 2) is characterized by a columnar arrangement of molecules extending in the direction of the crystallographic *b*-axis. Within the molecular columns only one of the aromatic rings (designated as A in Fig. 1) of related molecules are involved in "face-to-face" interactions with a centroid···centroid distance of 3.81 (1) Å between such rings.

### Crystal data

**Table:**

C_12_H_12_N_2_	*F*_000_ = 784
*M**_r_* = 184.24	*D*_x_ = 1.280 Mg m^−^^3^
Orthorhombic, *P**b**c**a*	Mo *K*α radiation λ = 0.71073 Å
Hall symbol: -P 2ac 2ab	Cell parameters from 5227 reflections
*a* = 11.7961 (3) Å	θ = 2.6–31.2º
*b* = 7.6130 (2) Å	µ = 0.08 mm^−^^1^
*c* = 21.2977 (5) Å	*T* = 153 (2) K
*V* = 1912.61 (8) Å^3^	Needle, colourless
*Z* = 8	0.54 × 0.24 × 0.14 mm

### Data collection

**Table:**

Bruker X8 APEXII CCD area-detector diffractometer	3233 independent reflections
Radiation source: fine-focus sealed tube	2262 reflections with *I* > 2σ(*I*)
Monochromator: graphite	*R*_int_ = 0.066
*T* = 153(2) K	θ_max_ = 31.7º
φ and ω scans	θ_min_ = 2.6º
Absorption correction: multi-scan(SADABS; Sheldrick, 2003)	*h* = −17→17
*T*_min_ = 0.924, *T*_max_ = 0.989	*k* = −10→11
26242 measured reflections	*l* = −31→28

### Refinement

**Table:**

Refinement on *F*^2^	Secondary atom site location: difference Fourier map
Least-squares matrix: full	Hydrogen site location: inferred from neighbouring sites
*R*[*F*^2^ > 2σ(*F*^2^)] = 0.045	H-atom parameters constrained
*wR*(*F*^2^) = 0.139	*w* = 1/[σ^2^(*F*_o_^2^) + (0.0856*P*)^2^] where *P* = (*F*_o_^2^ + 2*F*_c_^2^)/3
*S* = 0.91	(Δ/σ)_max_ < 0.001
3233 reflections	Δρ_max_ = 0.37 e Å^−^^3^
129 parameters	Δρ_min_ = −0.25 e Å^−^^3^
Primary atom site location: structure-invariant direct methods	Extinction correction: none

### Fractional atomic coordinates and isotropic or equivalent isotropic displacement parameters (Å^2^)

**Table:**

	*x*	*y*	*z*	*U*_iso_*/*U*_eq_	
N1	0.83087 (7)	0.16449 (12)	0.48415 (4)	0.0253 (2)	
N2	0.38746 (7)	−0.09210 (12)	0.27478 (4)	0.0235 (2)	
C1	0.65454 (8)	0.06887 (12)	0.40087 (5)	0.0179 (2)	
C2	0.76889 (8)	0.05080 (13)	0.38471 (5)	0.0206 (2)	
H2	0.7889	0.0062	0.3446	0.025*	
C3	0.85371 (8)	0.09788 (13)	0.42717 (5)	0.0215 (2)	
C4	0.72102 (9)	0.18301 (15)	0.49892 (5)	0.0269 (2)	
H4	0.7034	0.2310	0.5389	0.032*	
C5	0.63117 (8)	0.13748 (14)	0.46021 (5)	0.0230 (2)	
H5	0.5551	0.1527	0.4738	0.028*	
C6	0.97634 (9)	0.07391 (15)	0.41063 (6)	0.0278 (2)	
H6A	1.0152	0.1875	0.4127	0.042*	
H6B	0.9825	0.0265	0.3680	0.042*	
H6C	1.0115	−0.0079	0.4404	0.042*	
C7	0.56279 (8)	0.01492 (12)	0.35706 (5)	0.0175 (2)	
C8	0.44859 (8)	0.05087 (13)	0.36967 (5)	0.0194 (2)	
H8	0.4285	0.1139	0.4065	0.023*	
C9	0.36413 (8)	−0.00544 (13)	0.32832 (5)	0.0206 (2)	
C10	0.49692 (8)	−0.12586 (15)	0.26316 (5)	0.0247 (2)	
H10	0.5148	−0.1876	0.2257	0.030*	
C11	0.58594 (8)	−0.07721 (13)	0.30182 (5)	0.0220 (2)	
H11	0.6618	−0.1061	0.2909	0.026*	
C12	0.24104 (9)	0.02673 (16)	0.34184 (6)	0.0288 (3)	
H12A	0.2111	0.1128	0.3119	0.043*	
H12B	0.2327	0.0721	0.3847	0.043*	
H12C	0.1990	−0.0837	0.3378	0.043*	

### Atomic displacement parameters (Å^2^)

**Table:**

	*U*^11^	*U*^22^	*U*^33^	*U*^12^	*U*^13^	*U*^23^
N1	0.0222 (4)	0.0285 (5)	0.0252 (5)	0.0012 (3)	−0.0059 (4)	−0.0026 (3)
N2	0.0210 (4)	0.0277 (5)	0.0218 (5)	−0.0023 (3)	−0.0015 (3)	−0.0032 (3)
C1	0.0165 (4)	0.0192 (4)	0.0179 (5)	−0.0005 (3)	−0.0014 (3)	0.0009 (3)
C2	0.0178 (4)	0.0245 (5)	0.0196 (5)	−0.0002 (3)	−0.0006 (4)	−0.0001 (3)
C3	0.0185 (4)	0.0220 (5)	0.0241 (5)	−0.0006 (3)	−0.0034 (4)	0.0030 (4)
C4	0.0250 (5)	0.0343 (6)	0.0214 (5)	0.0027 (4)	−0.0038 (4)	−0.0068 (4)
C5	0.0198 (4)	0.0290 (5)	0.0201 (5)	0.0010 (4)	−0.0012 (4)	−0.0027 (4)
C6	0.0173 (5)	0.0353 (6)	0.0310 (6)	0.0003 (4)	−0.0031 (4)	0.0008 (4)
C7	0.0164 (4)	0.0191 (4)	0.0170 (5)	−0.0013 (3)	−0.0009 (3)	0.0021 (3)
C8	0.0172 (4)	0.0221 (5)	0.0189 (5)	0.0002 (3)	−0.0005 (3)	−0.0017 (3)
C9	0.0173 (4)	0.0230 (5)	0.0214 (5)	−0.0002 (3)	−0.0018 (4)	0.0001 (4)
C10	0.0230 (5)	0.0313 (5)	0.0197 (5)	−0.0019 (4)	0.0011 (4)	−0.0053 (4)
C11	0.0182 (4)	0.0281 (5)	0.0196 (5)	−0.0006 (3)	0.0012 (4)	−0.0025 (4)
C12	0.0176 (5)	0.0371 (6)	0.0316 (6)	0.0027 (4)	−0.0023 (4)	−0.0076 (5)

### Geometric parameters (Å, °)

**Table:**

N1—C4	1.3408 (13)	C6—H6B	0.9800
N1—C3	1.3426 (14)	C6—H6C	0.9800
N2—C10	1.3395 (13)	C7—C11	1.3967 (14)
N2—C9	1.3459 (13)	C7—C8	1.4006 (13)
C1—C5	1.3949 (14)	C8—C9	1.3971 (14)
C1—C2	1.3989 (13)	C8—H8	0.9500
C1—C7	1.4868 (13)	C9—C12	1.5004 (13)
C2—C3	1.3955 (14)	C10—C11	1.3848 (14)
C2—H2	0.9500	C10—H10	0.9500
C3—C6	1.5000 (14)	C11—H11	0.9500
C4—C5	1.3869 (14)	C12—H12A	0.9800
C4—H4	0.9500	C12—H12B	0.9800
C5—H5	0.9500	C12—H12C	0.9800
C6—H6A	0.9800		
			
C4—N1—C3	116.47 (9)	H6B—C6—H6C	109.5
C10—N2—C9	116.59 (9)	C11—C7—C8	116.59 (9)
C5—C1—C2	116.77 (9)	C11—C7—C1	121.67 (9)
C5—C1—C7	121.88 (9)	C8—C7—C1	121.73 (9)
C2—C1—C7	121.35 (9)	C9—C8—C7	120.34 (9)
C3—C2—C1	120.44 (10)	C9—C8—H8	119.8
C3—C2—H2	119.8	C7—C8—H8	119.8
C1—C2—H2	119.8	N2—C9—C8	122.59 (9)
N1—C3—C2	122.61 (9)	N2—C9—C12	116.14 (9)
N1—C3—C6	116.85 (9)	C8—C9—C12	121.27 (9)
C2—C3—C6	120.53 (10)	N2—C10—C11	124.74 (10)
N1—C4—C5	124.94 (10)	N2—C10—H10	117.6
N1—C4—H4	117.5	C11—C10—H10	117.6
C5—C4—H4	117.5	C10—C11—C7	119.13 (9)
C4—C5—C1	118.76 (10)	C10—C11—H11	120.4
C4—C5—H5	120.6	C7—C11—H11	120.4
C1—C5—H5	120.6	C9—C12—H12A	109.5
C3—C6—H6A	109.5	C9—C12—H12B	109.5
C3—C6—H6B	109.5	H12A—C12—H12B	109.5
H6A—C6—H6B	109.5	C9—C12—H12C	109.5
C3—C6—H6C	109.5	H12A—C12—H12C	109.5
H6A—C6—H6C	109.5	H12B—C12—H12C	109.5
			
C5—C1—C2—C3	0.87 (14)	C5—C1—C7—C8	8.36 (15)
C7—C1—C2—C3	−178.28 (9)	C2—C1—C7—C8	−172.52 (9)
C4—N1—C3—C2	0.45 (15)	C11—C7—C8—C9	0.41 (14)
C4—N1—C3—C6	−179.03 (9)	C1—C7—C8—C9	−178.39 (9)
C1—C2—C3—N1	−1.18 (15)	C10—N2—C9—C8	1.31 (15)
C1—C2—C3—C6	178.28 (9)	C10—N2—C9—C12	−178.16 (9)
C3—N1—C4—C5	0.55 (16)	C7—C8—C9—N2	−1.35 (15)
N1—C4—C5—C1	−0.80 (17)	C7—C8—C9—C12	178.10 (9)
C2—C1—C5—C4	0.05 (15)	C9—N2—C10—C11	−0.42 (17)
C7—C1—C5—C4	179.20 (9)	N2—C10—C11—C7	−0.46 (17)
C5—C1—C7—C11	−170.38 (9)	C8—C7—C11—C10	0.43 (14)
C2—C1—C7—C11	8.74 (14)	C1—C7—C11—C10	179.24 (9)

### Hydrogen-bond geometry (Å, °)

**Table:**

*D*—H···*A*	*D*—H	H···*A*	*D*···*A*	*D*—H···*A*
C6—H6C···N1^i^	0.98	2.73	3.6728 (16)	161

Symmetry codes: (i) −*x*+2, −*y*, −*z*+1.
